# Exploring the Interplay between COVID-19 and Gut Health: The Potential Role of Prebiotics and Probiotics in Immune Support

**DOI:** 10.3390/v16030370

**Published:** 2024-02-27

**Authors:** Marta Giovanetti, Gianfranco Pannella, Annamaria Altomare, Giulia Rocchi, Michele Guarino, Massimo Ciccozzi, Elisabetta Riva, Giovanni Gherardi

**Affiliations:** 1 Sciences and Technologies for Sustainable Development and One Health, Università Campus Bio-Medico di Roma, 00128 Roma, Italy; g.pannella@unicampus.it (G.P.); annamaria.altomare81@gmail.com (A.A.); 2 Climate Amplified Diseases and Epidemics (CLIMADE), Brasilia 70070-130, Brazil; 3 Instituto Rene Rachou, Fundação Oswaldo Cruz, Belo Horizonte 30190-002, Brazil; 4 Department of Agricultural, Enviromental and Food Science, University of Molise, 86100 Campobasso, Italy; 5 Research Unit of Gastroenterology, Università Campus Bio-Medico di Roma, 00128 Rome, Italy; giulia.rocchi@unicampus.it (G.R.); m.guarino@policlinicocampus.it (M.G.); 6 Operative Research Unit of Gastroenterology, Fondazione Policlinico Universitario Campus Bio-Medico, 00128 Rome, Italy; 7 Unit of Medical Statistics and Molecular Epidemiology, University Campus Bio-Medico of Rome, 00128 Roma, Italy; m.ciccozzi@unicampus.it; 8 Unit of Virology, Fondazione Policlinico Universitario Campus Bio-Medico, 00128 Rome, Italy; e.riva@unicampus.it; 9 Applied Bacteriological Sciences Unit, Department of Medicine and Surgery, Università Campus Bio-Medico di Roma, 00128 Rome, Italy

**Keywords:** probiotics, prebiotics, gut microbiota, immune system, viral infections, COVID-19

## Abstract

The COVID-19 pandemic has profoundly impacted global health, leading to extensive research focused on developing strategies to enhance outbreak response and mitigate the disease’s severity. In the aftermath of the pandemic, attention has shifted towards understanding and addressing long-term health implications, particularly in individuals experiencing persistent symptoms, known as long COVID. Research into potential interventions to alleviate long COVID symptoms has intensified, with a focus on strategies to support immune function and mitigate inflammation. One area of interest is the gut microbiota, which plays a crucial role in regulating immune responses and maintaining overall health. Prebiotics and probiotics, known for their ability to modulate the gut microbiota, have emerged as potential therapeutic agents in bolstering immune function and reducing inflammation. This review delves into the intricate relationship between long COVID, the gut microbiota, and immune function, with a specific focus on the role of prebiotics and probiotics. We examine the immune response to long COVID, emphasizing the importance of inflammation and immune regulation in the persistence of symptoms. The potential of probiotics in modulating immune responses, including their mechanisms in combating viral infections such as COVID-19, is discussed in detail. Clinical evidence supporting the use of probiotics in managing long COVID symptoms is summarized, highlighting their role as adjunctive therapy in addressing various aspects of SARS-CoV-2 infection and its aftermath.

## 1. Introduction

### 1.1. Background on the COVID-19 Pandemic and Its Impact on Global Health

The COVID-19 pandemic, caused by the severe acute respiratory syndrome coronavirus 2 (SARS-CoV-2), has dramatically impacted global health, leading to extensive illness, death, and significant societal and economic disruptions [1]. First identified in Wuhan, China, in December 2019, the SARS-CoV-2 is a novel coronavirus and part of a larger family that includes pathogens causing mild conditions like the common cold as well as severe diseases such as Middle East Respiratory Syndrome (MERS) and Severe Acute Respiratory Syndrome (SARS) [2]. Transmission primarily occurs through respiratory droplets during coughing, sneezing, or talking, though contact with contaminated surfaces is also a possible, albeit secondary, route [3,4].

The symptoms of COVID-19 vary widely, ranging from mild respiratory issues to severe complications like pneumonia, acute respiratory distress syndrome, organ failure, and death, especially in older individuals and those with pre-existing conditions [5]. Common symptoms include fever, cough, and breathing difficulties. Uniquely, the virus can also cause atypical symptoms affecting various body systems, such as gastrointestinal infections, diarrhea, and ulcerative colitis. Notably, patients with gastrointestinal manifestations often experience more severe respiratory complications [6], possibly due to a microbial imbalance characterized by a reduction in beneficial bacteria like *Lactobacillus* and *Bifidobacterium* [7].

**Figure 1:**
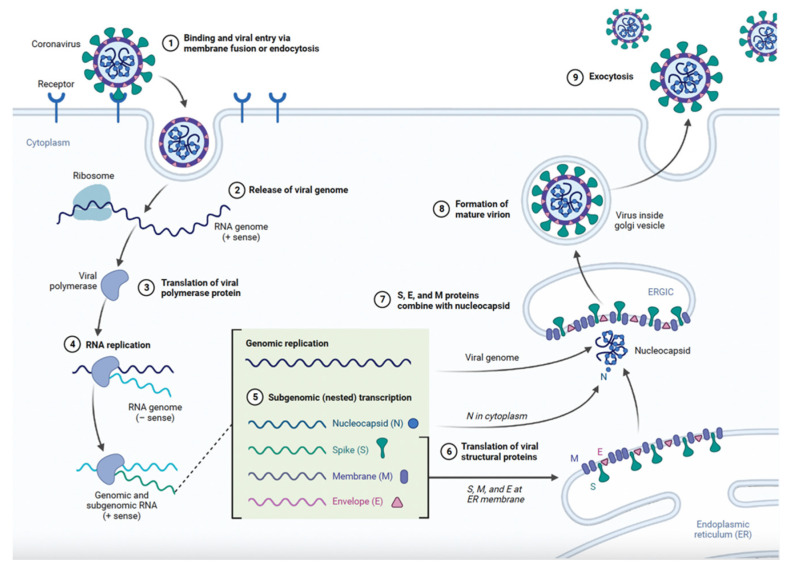
SARS-CoV-2 life cycle.

The pathogenesis of COVID-19 begins when the SARS-CoV-2 virus binds to angiotensin-converting enzyme 2 (ACE2) receptors found on human cells, predominantly in the lungs, heart, kidneys, and intestines (Figure 1) [8]. This interaction is critical to understanding the disease’s progression and its diverse impact on human health.

The virus’s spike protein plays a crucial role in facilitating its binding to host cells. Upon attachment, the virus fuses with the host cell membrane, a process potentially aided by cellular enzymes, leading to the release of its RNA into the cytoplasm [9]. The host cell then employs its own mechanisms to translate this viral RNA into two large polypeptides, which the virus’s proteases further cleave into smaller, functional proteins. Among these, some form the replication–transcription complex (RTC), vital for the replication of viral RNA. Subsequently, the newly synthesized viral RNA and structural proteins are transported to the endoplasmic reticulum and Golgi apparatus, where they assemble into new virus particles [9]. These virions then bud into the lumen of the ER–Golgi intermediate compartment (ERGIC), get encased in vesicles, and are finally released from the host cell through exocytosis [9]. The virus triggers an immune response which is essential for combating the infection but can also cause tissue damage. In severe cases, this response may become hyperactive, leading to a cytokine storm, potentially resulting in acute respiratory distress syndrome (ARDS) and multi-organ failure [10]. Understanding these mechanisms is key in developing effective treatments and preventative strategies. Beyond the immediate health impact, COVID-19 has profoundly affected global healthcare systems, disrupted routine medical services, and exerted a psychological and socioeconomic toll due to containment measures like lockdowns and travel restrictions. The pandemic has highlighted the interconnectedness of global health and the importance of international collaboration in facing such challenges.

### 1.2. Overview of the Immune Response to COVID-19 and the Role of Inflammation

The immune response to COVID-19 plays a crucial role in determining the course and severity of the disease [11]. When the body is infected by SARS-CoV-2, it triggers an immune response starting with the innate immune system, the body’s first line of defense [12]. This system includes physical barriers such as the skin and mucosal linings and immune cells like macrophages and dendritic cells that respond to pathogens in a non-specific way [13]. The presence of the virus activates these innate immune cells, which attempt to eliminate it through processes like phagocytosis and the release of various cytokines. These cytokines are critical in managing the infection but can also lead to inflammation [14]. SARS-CoV-2 has developed mechanisms to evade immune detection, including reducing the interferon (IFN) response, leading to lower levels of type I and II IFNs and IFN-stimulated genes (ISGs) in the early stages of infection [14]. IFNs typically help clear infections by promoting ISG transcription and producing antiviral proteins and cytokines. However, in severe COVID-19 cases, dysregulated cytokine and IFN feedback loops can exacerbate the cytokine storm, resulting in hyperinflammation, multi-organ failure, and death [11,12,13,14]. Following the innate response, the adaptive immune system kicks in, offering a more targeted attack against the virus. This includes B cells producing antibodies to neutralize the virus and prevent cell invasion. T cells, another component of the adaptive system, can destroy virus-infected cells and help coordinate the immune response. A key factor in severe COVID-19 cases is the overproduction of cytokines, leading to the notorious ‘cytokine storm’ [15]. This excessive inflammatory response can cause significant tissue damage, ARDS, and multi-organ failure [16], contributing to the high morbidity and mortality rates in severe COVID-19 cases. Moreover, the immune response to COVID-19 varies significantly among individuals. Some exhibit efficient responses, eliminating the virus with minimal symptoms, while others have delayed or inadequate responses, leading to severe illness. The reasons for this variability are not fully understood but may include genetic factors, pre-existing health conditions, age, and possibly previous exposure to other coronaviruses (though this aspect remains under debate). Understanding the intricate details of the immune response to COVID-19, particularly the role of inflammation, is vital in developing effective treatments and preventive strategies.

### 1.3. Overview of the Importance of the Immune System and Gut Microbiota in Maintaining Health

The immune system and gut microbiota are fundamental in maintaining overall health, each playing a critical role in disease prevention and normal bodily functions [17]. The immune system functions as the body’s protective mechanism against pathogens and other potential dangers. The immune system carries out a sequence of actions called the immune response to combat invading organisms and substances that infiltrate the body and lead to sickness [18]. This system comprised an intricate network of cells, tissues, and organs working together to protect the organism. Central to the immune response are leukocytes, or white blood cells, which are categorized into two main types: innate immune cells, the body’s first line of defense, and adaptive immune cells, which provide a more specific response to particular pathogens [17,18,19]. The gut microbiota, consisting of a diverse array of microorganisms like bacteria, viruses, fungi, and protozoa inhabiting the gastrointestinal system, significantly influences the body’s immune response. An optimal gut microbiota is crucial for immune system maturation, maintaining its balance, and protecting the host from harmful microbes [19]. These microorganisms aid in immune cell development, the production of antimicrobial substances, nutrient breakdown and absorption, vitamin synthesis, and the generation of short-chain fatty acids, vital for gut health [17,18,19]. The interaction between gut bacteria and the immune system is complex and ongoing, involving a mutual exchange where the microbiota helps maintain tolerance towards beneficial microbes and enables the immune response against pathogens. This communication is facilitated by microbial-associated molecular patterns (MAMPs) recognized by pattern recognition receptors (PRRs) on immune cells [20]. Dysbiosis, or an imbalance in the gut microbiota composition, has been linked to various conditions, including inflammatory bowel disease, functional gastrointestinal disorders, viral infections, allergies, obesity, and even mental health issues [21]. Thus, a healthy gut microbiota is crucial not just for gastrointestinal health but also for broader health implications. Maintaining a healthy gut involves mindful dietary choices, considering lifestyle factors like physical activity, smoking, and alcohol consumption, and avoiding unnecessary antibiotic use, which can disrupt microbial balance [21]. Probiotics and prebiotics are used to improve gut microbiota composition, potentially enhancing immune function and overall health [21].

## 2. Prebiotics and Probiotics

Clear definitions are vital for terms such as ‘probiotic’, ‘prebiotic’, ‘synbiotic’, and other terms recently introduced in the functional foods’ domain [22,23]. Probiotics, a key focus in this field, are live microorganisms that, when consumed in adequate amounts, confer health benefits to the host. Werner Kollath was the first to use the term ‘probiotic’ in 1953, defining it as organisms that significantly contribute to health improvement [23,24,25]. The FAO and WHO describe probiotics as live microorganisms that, when ingested in sufficient quantities, have a positive impact on the host’s health. The ISAPP defines a prebiotic as ‘a substrate selectively utilized by host microorganisms conferring a health benefit’ [26]. To be classified as a prebiotic, a substance must be resistant to gastric acidity, hydrolysis by mammalian enzymes, and gastrointestinal absorption; fermentable by the gut microbiota; and selectively stimulate the growth and/or activity of gut bacteria linked to health [26]. Although the FDA has not officially recognized the term ‘prebiotic’, many substances identified as prebiotics by the ISAPP are categorized by the FDA as ‘non-digestible carbohydrates with a physiological effect’ [26,27,28,29]. These include various fibers, such as non-fiber prebiotics (e.g., cellulose, pectin, guar gum, psyllium husk) and fiber prebiotics like inulin, inulin-type fructans (ITF), and galacto-oligosaccharides (GOS) [30,31]. Recent years have seen the emergence of other substances like lactulose being recognized as non-fiber prebiotics or prebiotic candidates (Table 1). While prebiotics are predominantly known for enhancing beneficial bacteria like bifidobacteria and lactobacilli, the research on their broader effects on the gut microbiota in humans and animals is still developing. Studies on substances such as galacto- and fructo-oligosaccharides have demonstrated their potential antidepressant and anxiolytic properties, including their ability to mitigate the effects of chronic stress [32].

Furthermore, new terms such as synbiotics, postbiotics, and paraprobiotics have emerged in this field. Synbiotics, which combine prebiotics and probiotics, work to enhance the viability and efficacy of probiotic microorganisms, thereby boosting the presence of beneficial microbes in the gastrointestinal tract. Studies suggest that synbiotics can be more effective than using either probiotics or prebiotics alone [33,34,35]. Postbiotics, also referred to as metabiotics, biogenics, or cell-free supernatants (CFSs), are composed of metabolic byproducts and soluble factors produced during bacterial fermentation. These include short-chain fatty acids (SCFAs), enzymes, antimicrobial peptides (AMPs), teichoic acids, endo- and exopolysaccharides, cell surface proteins, vitamins, plasmalogens, organic acids, and other bioactive compounds, originating from live or lysed bacteria [35]. Paraprobiotics, distinct in their category, are nonviable or inactivated microbial cells that, when administered in adequate quantities, can induce beneficial biological activities in the host [35].

### 2.1. Probiotics and Their Role in Gut Microbiota and Immune System

**Figure 2:**
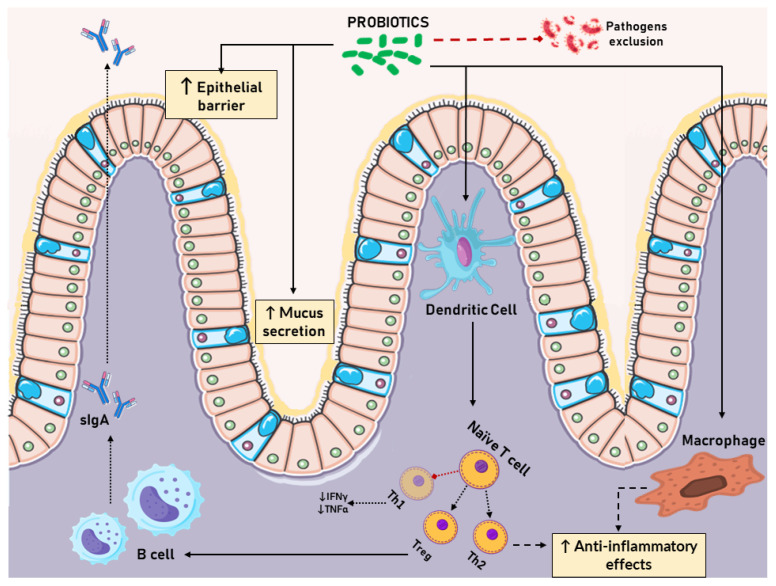
The role of probiotics in gut homeostasis.

The complex link between microbiota composition and human health is evident from the significant variations in gut microbiota observed between healthy individuals and those with various diseases and pathologies. These differences have implications not just for the intestine but also for mucosal tissues and the entire body [36,37]. Probiotics are key in modulating the gut microbiota and the immune system (Figure 2). Their roles include exerting antiallergic effects, anticancer activities, influencing inflammatory intestinal diseases, and impacting neurological disorders [38]. The metabolites produced by probiotics are gaining recognition for their importance in facilitating host–microbe interactions, which can be nutritionally influenced. These metabolites can improve intestinal barrier function, promote gastric motility and hormonal secretion, offer anti-inflammatory and antioxidant benefits, synthesize neurotransmitters, and alter the gut microbiome’s composition and metabolism [38,39,40]. Additionally, probiotics can prevent pathogens from adhering to mucosal cells and modulate the host’s immune response, further strengthening the intestinal barrier [41].

Strategies like probiotic administration are employed to correct dysbiosis in the gut microbiome, aiming to restore its equilibrium [42]. Probiotics can increase microbial diversity, boost lactase enzyme production, improve the immune microenvironment, and enhance intestinal permeability [43,44]. The human gastrointestinal tract harbors a vast array of symbiotic bacteria, forming a dynamic ecosystem with substantial physiological influence [45]. This microbiome plays a critical role in early life, where initial microbial colonization can shape the future development of allergies, obesity, and inflammatory conditions [46,47]. The establishment of gut microbiota in infancy is crucial for the development of the mucosal immune system [48]. Influences such as delivery method and breast milk composition can impact neonatal microbial colonization and mucosal immunity development, with long-term effects on immune-mediated diseases [49]. The timing of bacterial colonization in early life is pivotal for proper immune development, and ongoing research is illuminating aspects of maternal-to-infant microbial transmission during pregnancy and childbirth [50]. Intriguingly, the human placenta contains DNA from probiotic Bifidobacteria and Lactobacilli, while exposure to pathogenic bacteria like *Ureaplasma* species can disrupt immune development, leading to significant complications [51]. The mucosal immune system and commensal bacteria have co-evolved, particularly at mucosal sites, to maintain a balance between stability and pathogen defense [52]. Key immune cells like dendritic cells and macrophages, found throughout the body, regulate immune responses and maintain tissue homeostasis [53]. Innate lymphoid cells (ILCs), abundant in mucosal tissues and similar to phagocytes, interact with commensal microbiota, affecting cytokine production and various immune functions, highlighting their role in mucosal infection responses [54]. The host’s immune system shapes the microbiota, especially through secretory immunoglobulin A (sIgA), which binds to the gut microbiota, mediating effects like immune exclusion and microbial growth modulation [55]. The microbiome’s role in developing atopic diseases is well established [56]. Probiotics and their derivatives stimulate the immune system and modulate immunological responses, affecting dendritic cells, macrophages, B cells, and T cells [57]. They promote anti-inflammatory cytokine production, interact with gut–brain-axis neurotransmitters, and influence stress-related pathways [58,59,60]. The benefits of probiotics extend to reducing nutritional intolerances, improving nutrient availability, and alleviating allergies [61]. By targeting the gut microbiome, they manage allergy symptoms through modulating immune and inflammatory responses, believed to improve mucosal barrier functions and cytokine expression, thereby balancing Th1 and Th2 immune responses [62,63,64,65,66].

### 2.2. Probiotics and Their Roles on Viral Infections

**Figure 3:**
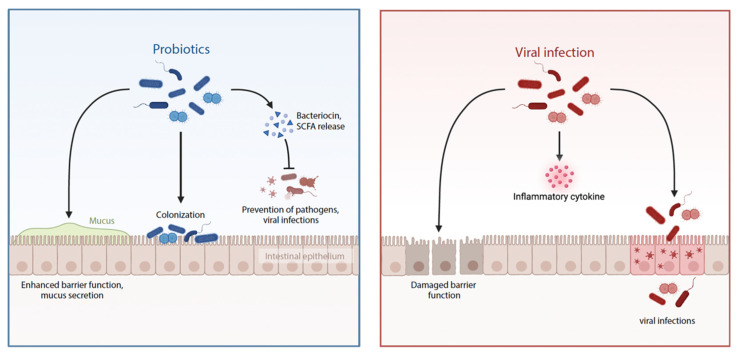
The role of probiotics in modulating viral infections.

Viral infectious diseases are a major contributor to the global burden of death and disability in both developed and developing countries [67,68]. A healthy and diverse microbial population, predominantly situated in the intestines, can protect the human host from various pathogenic diseases through a range of mechanisms, exerting substantial inhibitory effects [69]. Consequently, probiotics serve as a supportive approach, offering beneficial effects against viral infections by bolstering the immune response, maintaining the integrity of the protective cellular layer, and interacting with harmful microorganisms to impede their attachment (Figure 3).

The antiviral properties of specific *Lactobacillus* and *Bifidobacterium* strains against gastrointestinal and respiratory viruses have been extensively studied [70]. Numerous research studies highlight the effectiveness of probiotics as complementary treatments for rotavirus (RT), influenza virus, and human respiratory syncytial virus (RSV). Rotavirus (RV), a leading cause of severe diarrhea in children under five globally, alters the gut microbiome’s composition, shifting from Bacteroidetes to Firmicutes, which decreases bacterial diversity and increases the presence of harmful bacteria like Shigella [71]. The gastrointestinal (GI) tract, containing nearly 70% of the body’s immune cells, forms a critical link between the immune system and gut microorganisms, with notable interactions between the immune system and GI viruses. A recent study demonstrated that a combination of *Bifidobacterium longum* and *Chlorella sorokiniana* could enhance the cellular antiviral immune response [72]. Furthermore, *Lactobacillus* and *Bifidobacterium* have shown immunomodulatory effects against RV, both independently and in combination with RV vaccines, acting as immunostimulants [73,74,75,76]. Probiotics have been identified as beneficial adjuncts to RV vaccinations. Beyond GI viruses, there is growing evidence of probiotics and the gut microbiota exerting antiviral effects against respiratory viruses like the influenza virus [76,77]. Studies suggest that probiotics administered nasally or orally can bolster resistance to respiratory viral infections by stimulating the release of protective cytokines from alveolar macrophages and NK cells, crucial in defense mechanisms [78,79,80]. RSV, known for causing severe respiratory illness in infants and children, has a unique relationship with gut microbiota and probiotics [81,82,83]. Oral administration of specific *Lactobacillus* strains has been shown to significantly reduce RSV levels in the lungs by modulating the innate immune response in the respiratory system [84,85,86,87,88].

### 2.3. Probiotics and Their Role in COVID-19 Disease

Evidence suggests that post-COVID-19 recovery can be accompanied by intestinal microbial dysbiosis, a disruption in gut microbial balance lasting up to six months [89]. This dysbiosis, marked by a decrease in beneficial bacteria like *Lactobacillus* and *Bifidobacterium*, could hinder the recovery process in COVID-19 patients [90]. Reestablishing a balanced relationship between the lungs and the gut microbiota is thought to offer therapeutic advantages in combating COVID-19. Comprehending how probiotics or their metabolites exert their influence is essential for utilizing their potential in stabilizing the gut microbiota and potentially preventing or alleviating SARS-CoV-2 infection. Computational analyses, including drug discovery through computer modeling and machine learning predictive models, are crucial in the field of probiotics research. These tools help analyze extensive datasets and explore various mechanisms related to the microbiome and protein structures [90]. SARS-CoV-2 primarily targets the respiratory tract by binding to the ACE2 receptor, found in various organs, particularly type II alveolar and airway epithelial cells [90]. The virus also affects the gastrointestinal tract, causing symptoms like nausea, diarrhea, and vomiting, with its RNA detectable in fecal samples of infected individuals [91,92]. Compared to healthy counterparts, COVID-19 patients show a reduction in gut microbiota diversity, with a decrease in immunomodulatory commensals like *Eubacterium rectale* and *Bifidobacterium*. In contrast, genera such as *Collinsella*, *Streptococcus*, and *Morganella* are more abundant in these patients, along with species like *Coprobacillus*, *Clostridium ramosum*, and *Clostridium hathewayi*, particularly in those with a higher potential for SARS-CoV-2 transmission [93]. The gut–lung axis (GLA), which describes the bidirectional interaction between respiratory mucosa and gut microbiota, is pivotal for effective COVID-19 treatment by modulating the immune response [92]. The presence of specific microbial genera and species in the gut is associated with varying levels of SARS-CoV-2 infectivity, indicating the role of gut microbiota in immune enhancement through short-chain fatty acid production [94]. Healthy lungs also host a unique microbiota, including species of *Prevotella*, *Streptococcus*, *Veillonella*, *Fusobacterium*, and *Haemophilus* [95,96]. While the exact role of the microbiome in disease progression remains to be fully understood, there is a notable association between gut bacteria imbalances and increased susceptibility to pulmonary diseases [97]. This link is exemplified in conditions like inflammatory bowel disease (IBD), which are known to increase the risk of respiratory tract infections, highlighting the interplay between the lungs and the gut microbiota [98,99,100,101,102].

## 3. Probiotics as Adjuvant Treatment in COVID-19 Disease

Countries globally are grappling with the surge of infectious diseases, notably the COVID-19 pandemic, which has impacted millions. While therapeutic and preventive measures have shown success, the advent of new viral strains remains a concern. This situation highlights the necessity for innovative approaches to tackle viral infections that cause significant damage to organs such as the respiratory tract, liver, and colon, including the emerging challenge of long COVID [103,104].

In managing COVID-19, strategies like antiviral and anti-infective therapies are crucial but so is maintaining proper acid–base balance and restoring the microecological equilibrium [105]. A well-functioning intestinal ecosystem is key to defending against infections, and disturbances in nutritional and microecological balance can hinder the recovery of both intestinal health and lung function [84,85,86]. Patients with COVID-19 often exhibit microbial dysbiosis, characterized by a decline in *Lactobacillus* and *Bifidobacterium* levels in the gut [105]. Consequently, probiotics are being explored as a potential strategy against COVID-19 (Table 1) [106].

Research is increasingly focusing on probiotics with antiviral properties against the disease. Clinical trials have indicated that probiotics, including *Lactobacillus* acidophilus and *Bifidobacterium* infantis, may enhance immune function and reduce secondary infections in severe cases [107]. A study highlighted the effectiveness of a probiotic formula containing *Lactiplantibacillus plantarum* strains in improving COVID-19 outcomes in a controlled setting [108]. Additionally, diets rich in fermented vegetables have been linked to lower COVID-19 mortality rates [109,110]. Strains such as *Lactobacillus*, *Bifidobacterium* spp., *Leuconostoc mesenteroides*, and *Pediococcus pentosaceus* have shown promise in reducing the severity of COVID-19 [111], prompting the U.S. Food and Drug Administration to approve a microbiota-specific formula for early COVID-19 management in patients with obesity or type 2 diabetes [112].

The gut–lung axis is instrumental in understanding how probiotics might serve as adjunctive treatments for COVID-19, given their role in regulating both gut and lung environments. Meta-analyses have demonstrated that probiotics can prevent respiratory infections and reduce the incidence of acute respiratory infections without adverse effects [113,114], highlighting the interplay between the gastrointestinal tract’s immune and inflammatory state and other systems like the lungs [115,116]. Probiotic therapy in COVID-19 management focuses on restoring the gut microbiota’s diversity, composition, function, and metagenomic potential to healthy levels. Probiotics support the recovery of a healthy gut microbiota, bolster the intestinal barrier, and inhibit pathogen colonization [117,118,119,120,121]. They have been shown to modulate both innate and adaptive immune responses, enhancing the host’s immune response and improving gut flora [120,122,123,124,125]. Changes in immune cell levels, including naïve T helper cells and NK cells, have been observed in COVID-19 patients [126]. Clinical trials reveal that *Lactobacillus acidophilus* and *L. plantarum* can modulate cytokine release, providing immunomodulatory effects [124,127]. Omics techniques have identified probiotic-derived immunomodulatory genes and pathways, underscoring their role in cytokine regulation [128,129,130]. However, more research is essential to fully comprehend the extent of probiotics’ impact on maintaining gut immunological homeostasis in COVID-19 patients. A summary of studies on probiotics and their effects in COVD-19 infections is illustrated in Table 2.

## 4. Conclusions

Understanding the therapeutic roles of probiotics as adjunctive treatments, especially for emerging diseases like COVID-19 and long COVID, is becoming increasingly crucial. Detailed analysis and extensive research are vital in enhancing our comprehension of probiotics’ therapeutic effects. Furthermore, maintaining stringent production standards is essential for ensuring the quality and genetic stability of probiotic products. The application of probiotics in adjunctive COVID-19 and long COVID treatment has broadened their potential uses, enriching our knowledge of their function in the gut ecosystem and their interaction with the respiratory system. Longitudinal studies are necessary to illuminate the role of microbiota and the gut–lung axis in respiratory diseases, potentially leading to the use of specific probiotic strains in COVID-19 and long COVID treatment strategies. Over the past decade, significant advances have been made in understanding how the microbiota influences host immunity. Integrating technologies like artificial intelligence, machine learning, and computational studies with genomics- and omics-based analyses offers an efficient approach for the scientific community to further explore the host–microbiome relationship. Utilizing advancements in bioinformatics and computational research could unravel the molecular mechanisms through which probiotics affect SARS-CoV-2 and long COVID, bridging the divide between fundamental scientific discoveries and clinical practices, especially in modifying the microbiome to treat inflammatory diseases. Despite the availability of vaccines and widespread vaccination campaigns, the daily count of COVID-19 and long COVID cases remains high, continuing to impact human health and economies globally. Given the potential antiviral properties of probiotics and their byproducts, incorporating probiotics as an additional preventive measure alongside vaccines could represent a promising strategy to combat COVID-19 and long COVID.

## Figures and Tables

**Table 1:** Prebiotics and main prebiotic candidates.

Category	Sub-Category		Food Sources	References
Fiber prebiotics Non-digestible carbohydrates with physiological effect	Inulin and Inulin-type fructans (ITF)	Inulin (DP 2–60) Oligofructose (DP < 10) Short-chain fructooligosaccharides (scFOS) (DP 2–4)	Asparagus, leeks, garlic, chicory root, onion, Jerusalem artichoke, wheat, banana, agave Synthetized from sucrose	[26,27]
	Galagto-oligosaccharides (GOS)	Alpha-GOS Beta-GOS	Milk, produced enzymatically from lactose	[26,27,28,29,30]
Non-fiber prebiotics	Lactulose		Synthetic disaccharide from isomerization of lactose	[26]
Fiber candidate as prebiotics Non-digestible carbohydrates with physiological effect	Resistant starch (RS2)		Corn, potato, tapioca	[26]
	Polydextrose		Synthetic fiber	[26]
	Isomalto-oligosaccharides (IMOSs)	Isomaltose, panose, isomaltriose, isomaltotetraose, and isomaltopentose	Enzymatically produced from maltose and maltooligosaccharides	[26]
	Xylo-oligosaccharides (XOS) and Arabinoxylane (AXOS)		Cereals	[26]
	Human milk oligosaccharides (HMOs)	Neutral HMOs (e.g., 2′-Fucosyllactose (2′-FL) and Lactodifucopentaose Neutral N-containing HMOs (e.g., lacto-N-tetraose) Acids (HMOs) (e.g., 2′-sialyllactose)	Human milk	[26]
Non-fibers candidate as prebiotics	Polyphenol		Fruits and vegetables	[26]
	Polyunsaturated fatty acids		Crop seeds and vegetable oils, fish and fish oil	[26]
	Resistant proteins (RP)		Plant-based foods (e.g., soybean, buckwheat, rice, and potato), eggshell membrane	[26]

**Table 2:** Summary of studies on the effects of probiotics in COVID-19 and long COVID infection.

Type of Study/Method Features	Probiotic Tested	Main Results	Ref.
Computational study, molecular dynamics simulation	Plantaricin compounds, resulting from the metabolism of *Lacticaseibacillus plantarum.*	Plantaricin compounds—BN, JLA-9, W, D—showed antiviral activity, blocking the entry of SARS-CoV-2 by binding to RNA-dependent RNA polymerase (RdRp), the receptor-binding domain of SARS-CoV-2 (RBD) and angiotensin-converting enzyme 2 (ACE2).	[129]
Molecular docking analyses and molecular dynamics simulation	Metabolic compounds resulting from probiotic strains activity.	Glycocin F and Lactococcine G, derived from *Lactococcus lactis* and *Lactobacillus plantarum*, respectively, had high affinity for binding to viral proteins and could be administered as therapy to inhibit SARS-CoV-2 infection.	[130]
Molecular docking analyses and in silico experiments	Four probiotic-derived polypeptides: subtilisin, Curvacin A, Sakacin P, Lactococcin Gb.	Peptides derived from probiotic strains’ activity—Subtilisin (*Bacillus amyloliquefaciens*), Curvacin A (*Lactobacillus curvatus*), Sakacin P (*Lactobacillus sakei*), Lactococcin Gb (*Lactococcus lactis*)—showed a higher affinity to bind and block S-protein or RBD of S1 subunit of SARS-CoV-2 and human ACE2 receptor molecule.	[131].
Longitudinal cohort study [NCT04447144]	Commercial probiotic yogurt (PY) 1·4 × 10^9^ CFU of *Bifidum* bacteria	The evaluation of commercial PY intake in 170 patients with mild or moderate COVID-19 infection showed a significant negative correlation between PY intake and SARS-COV-2 infection severity. GI symptoms, such as diarrhea, were more frequent in COVID-19 patients who never ate PY than in those who consumed variable amounts.	[132]
Double-blind, RCT [NCT04366180]	*Lactobacillus coryniformis* K8 CECT 5711 (3 × 10^9^ CFU/day) vs. placebo (maltodextrin).	The administration of one capsule of *L. coryniformis* K8 per day helps to extend the immune protection generated by the COVID-19 vaccine over time.	[133]
Single-center, quadruple-blinded, RCT in adult symptomatic COVID-19 outpatients [NCT04517422]	Placebo vs. probiotic formula, *Lactiplantibacillus plantarum* KABP022, KABP023, KAPB033, *Pedicoccus acidilactici* KABP021 (2 × 10^9^ CFU), for 30 days.	Probiotic supplementation was well tolerated and reduced nasopharyngeal viral load, pulmonary infiltrates, and duration of digestive and nondigestive symptoms compared with placebo. No significant changes in fecal microbiota composition were detected between the probiotic and placebo. Supplementation with the probiotic significantly increased specific IgM and IgG against SARS-CoV-2.	[110,134]

## Data Availability

Not applicable.
